# Solitary Duodenal Ulcer Causing Biliary Obstruction Requiring Rendezvous Procedure in a Pediatric Patient With Eosinophilic Gastroenteritis

**DOI:** 10.7759/cureus.9377

**Published:** 2020-07-24

**Authors:** Jacquelin Peck, Kathryn M Kimsey, Evan Harris, Hector Monforte, Michael Wilsey

**Affiliations:** 1 Anesthesiology, Mount Sinai Medical Center, Miami Beach, USA; 2 Gastroenterology, Johns Hopkins All Children’s Hospital, St. Petersburg, USA; 3 Vascular and Interventional Radiology, Center for Vien Restoration, Glastonbury, USA; 4 Pathology, Johns Hopkins All Children’s Hospital, St. Petersburg, USA; 5 Gastroenterology, Johns Hopkins All Children's Hospital, St. Petersburg, USA; 6 Pediatrics, University of South Florida College of Medicine, Tampa, USA

**Keywords:** pediatrics, gastroenterology, endoscopy, biliary obstruction, eosinophilic gastroenteritis, endoscopic retrograde cholangiopancreatography, combined percutaneous-endoscopic rendezvous technique

## Abstract

Eosinophilic gastroenteritis (EGE) is an uncommon disease characterized by immune cell-mediated inflammation of the gastrointestinal (GI) tract resulting in vague abdominal symptoms, most commonly nausea, vomiting, diarrhea, and abdominal pain. We report the case of a 16-year-old male presenting with a six-week history of progressive pruritus, jaundice, fatigue, abdominal pain, acholic stools, and dark-colored urine. This patient was diagnosed via endoscopy with biliary obstruction caused by a large, solitary, duodenal ulcer secondary to EGE. This is a severe complication of EGE and to our knowledge, this is the first reported case of biliary obstruction caused by a duodenal ulcer in a pediatric patient with EGE. Additionally, we describe the first pediatric combined percutaneous-endoscopic rendezvous technique after failed therapeutic endoscopic retrograde cholangiography (ERCP) to relieve the biliary obstruction.

## Introduction

Eosinophilic gastroenteritis (EGE) is an immune-mediated disorder associated with peripheral eosinophilia and eosinophilic inflammation generally located in the stomach or duodenum. EGE may progress to mucosal ulceration, intestinal obstruction, and, in rare cases, perforation [1-4]. Biliary obstruction has been described in adults with peptic duodenal ulcers, but it occurs very rarely in EGE, and has yet to be described in the pediatric population [2-7]. Failure to relieve biliary obstruction with therapeutic endoscopic retrograde cholangiography (ERCP) may require alternative drainage modalities.

## Case presentation

A 16-year-old male with a history of reflux esophagitis and a duodenal ulcer diagnosed three years prior by upper endoscopy and treated with proton pump inhibitor therapy presented with a six-week history of progressive pruritus, jaundice, fatigue, abdominal pain, acholic stools, and dark-colored urine. Work-up revealed a total bilirubin 5.6 mg/dL, direct bilirubin 3.5 mg/dL, aspartate aminotransferase (AST) 43 U/L, alanine transaminase (ALT) 55 U/L, alkaline phosphatase 250 U/L, gamma-glutamyl transferase (GGT) 34 U/L, lipase 24 U/L, C-reactive protein (CRP) < 0.5 mg/L, and an elevated absolute eosinophil count (AEC) 1,000 eosinophils per microliter (<500) . Abdominal ultrasound (US) revealed a dilated common bile duct (1 cm), and magnetic resonance cholangiopancreatography (MRCP) showed gallbladder distention with marked intra- and extrahepatic biliary ductal dilatation without cholelithiasis or choledocholithiasis.

**Figure 1:**
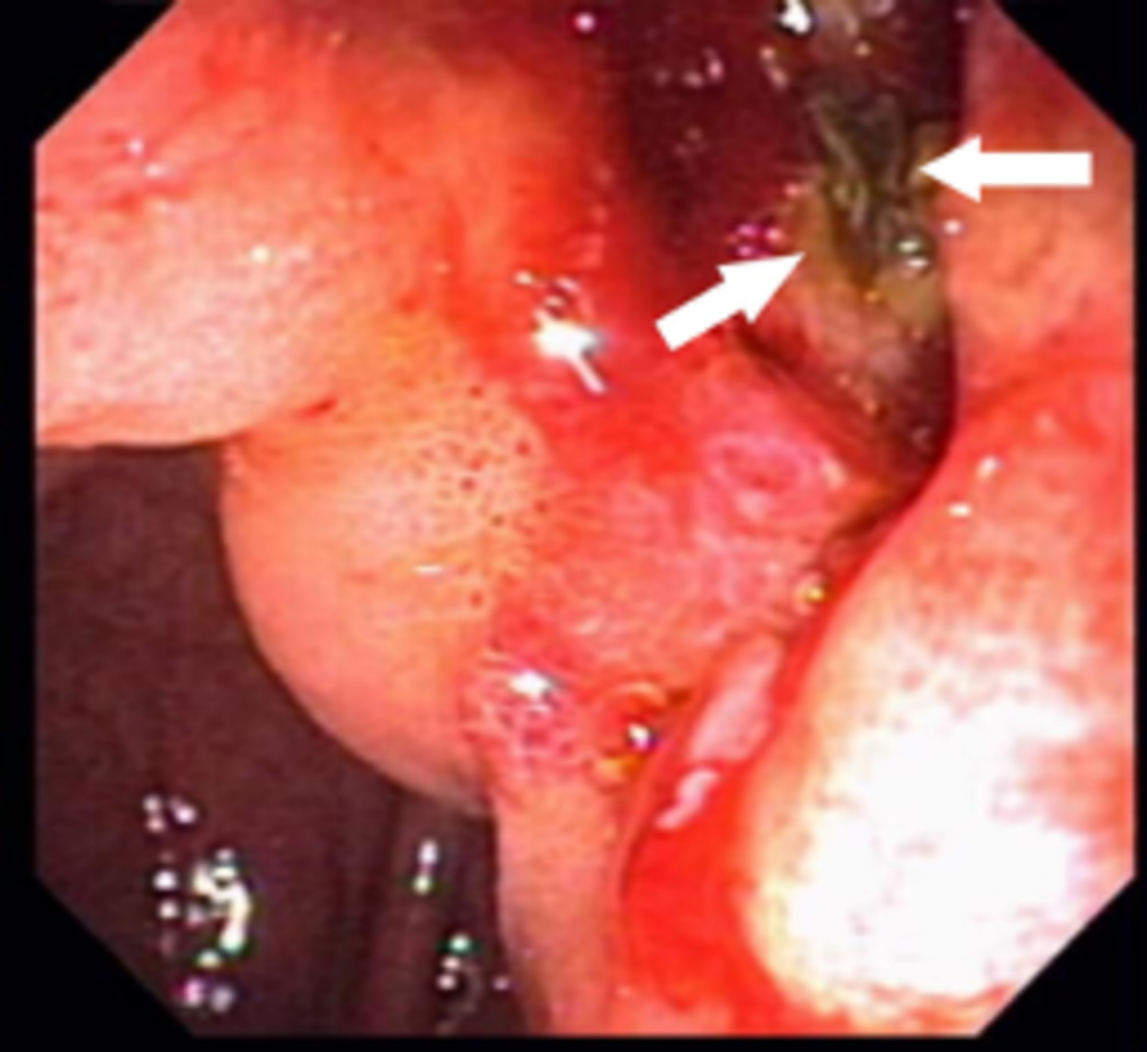
Duodenal ulcer, located just proximal to the ampulla of Vater. Debris (white arrows) is noted within the ulcer bed.

**Figure 2:**
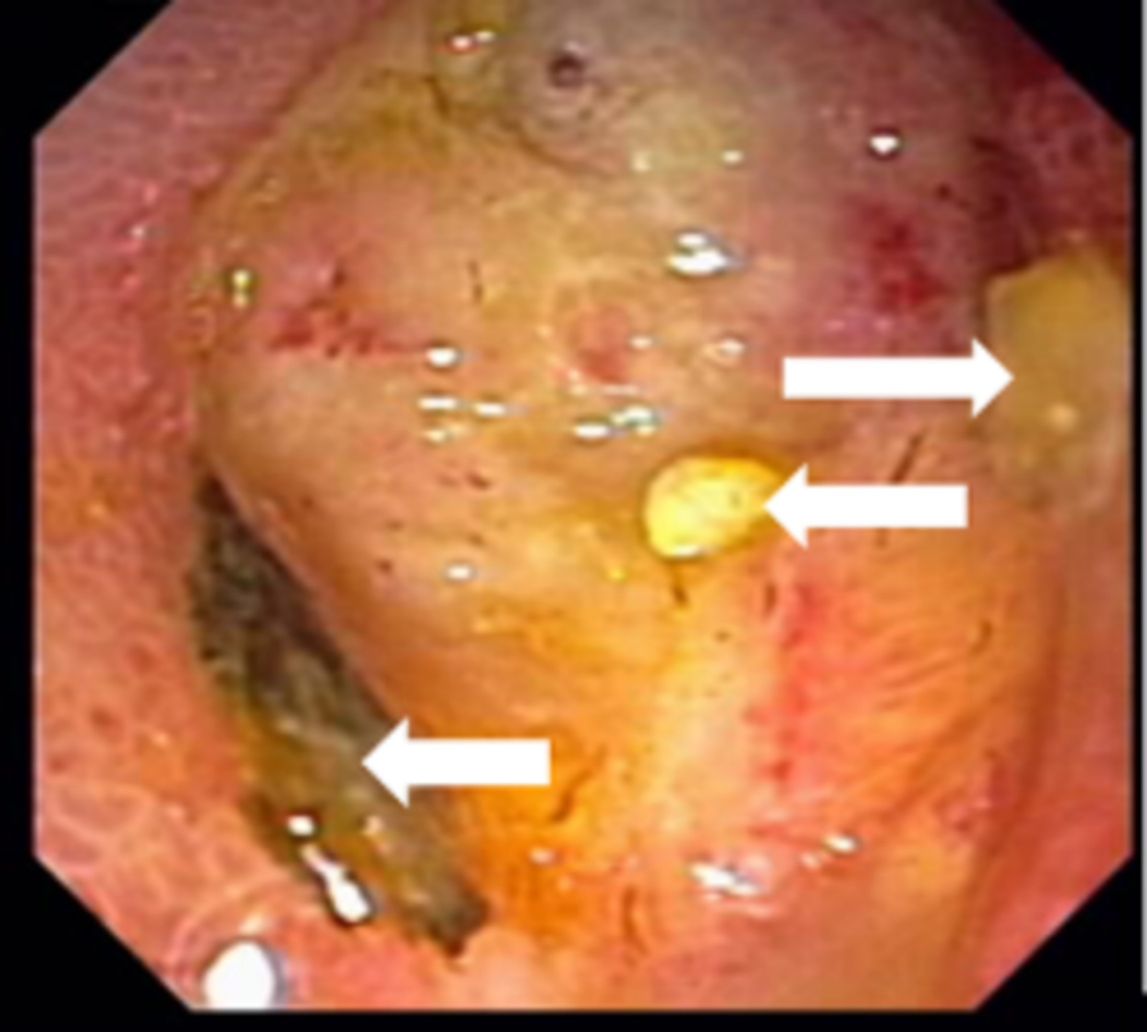
Duodenal ulcer bed (close-up view) showing no evidence of bleeding. Debris (white arrows) is noted within the ulcer bed.

ERCP identified a large duodenal ulcer on the lesser curvature of the duodenal sweep, just proximal to the ampulla of Vater (Figures 1, 2), which was not actively bleeding, did not require endotherapy, and was confirmed by upper esophagogastroduodenoscopy (EGD).

**Figure 3:**
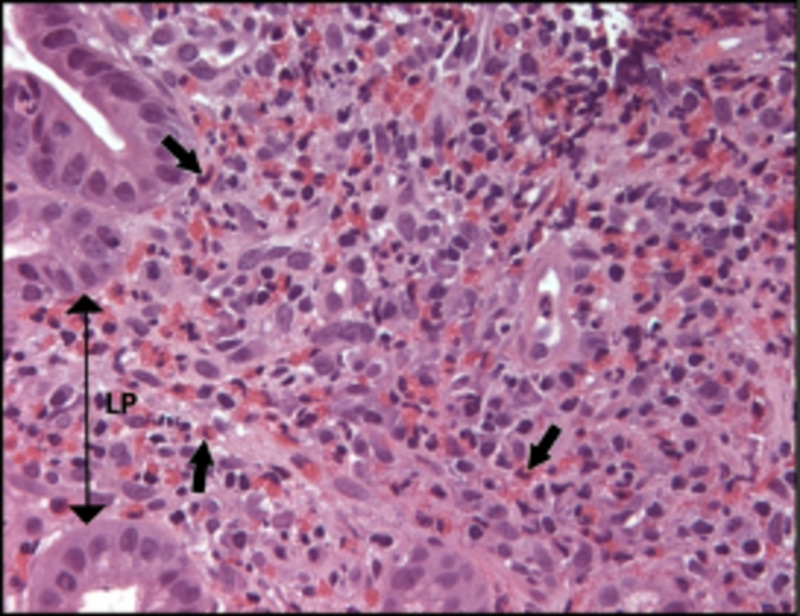
Photomicrograph of marked duodenal eosinophil infiltration expanding lamina propria (double headed arrow) and within crypts. There are 250+ eosinophils in this field (black arrows). Magnification x100.

Deep biliary cannulation and stent placement could not be performed due to biliary obstruction. Endoscopic mucosal biopsies revealed eosinophilic esophagitis, gastritis, and duodenitis (Figure 3). 

**Figure 4:**
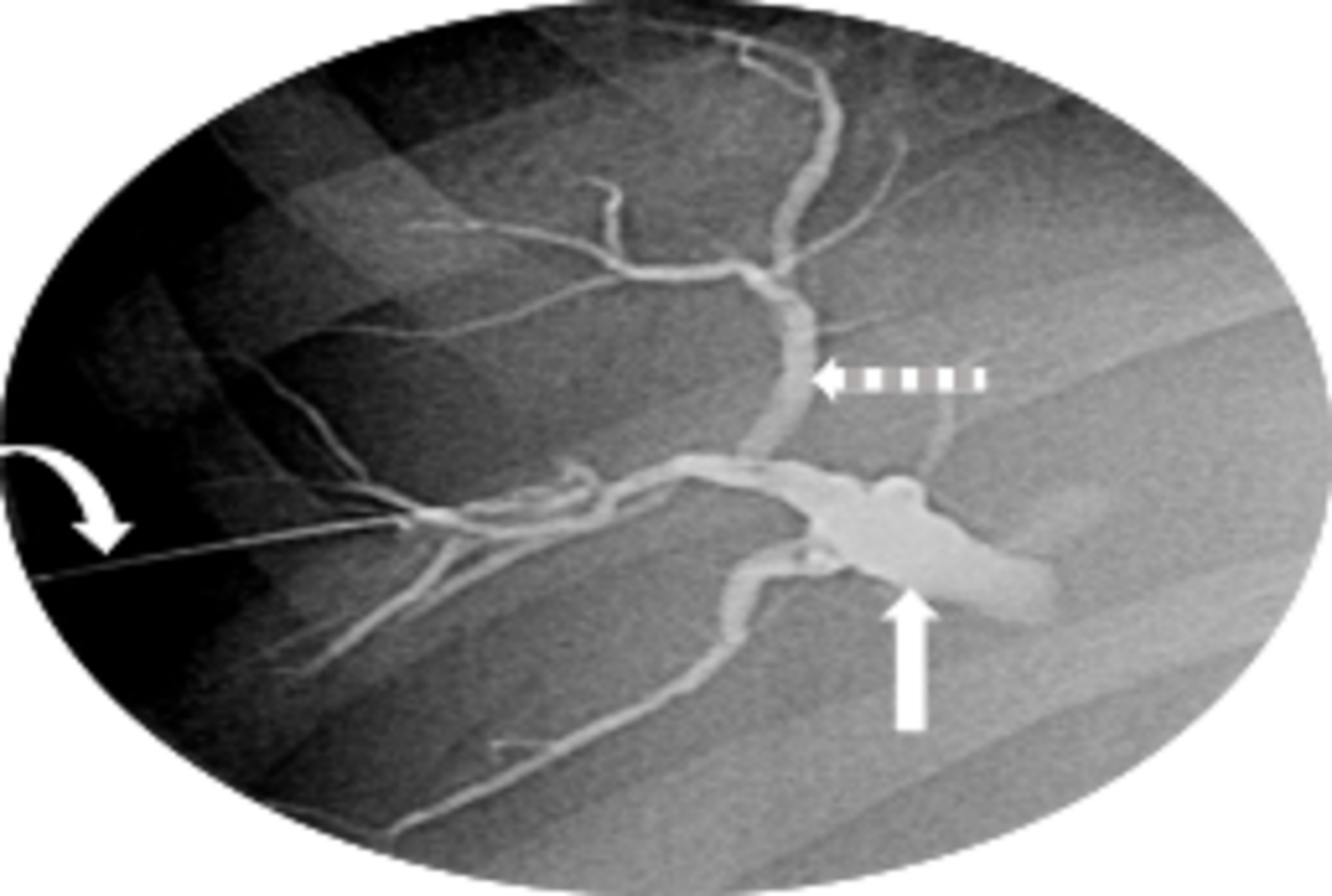
Percutaneous transhepatic cholangiography showing dilation of the intrahepatic bile ducts (dashed arrow) and common bile duct (straight arrow). Transhepatic needle shown (curved arrow, left).

Intravenous (IV) antibiotic therapy with 3.1 g of ticarcillin-clavulanate every six hours and 2 mg per kilogram of gentamicin every eight hours was initiated for cholangitis prophylaxis, and fluoroscopically guided percutaneous transhepatic biliary drainage (PTBD) (Figure 4) was performed by interventional radiology. One week later, the patient’s percutaneous drain was exchanged for a 10-French, 7-cm endoscopic biliary stent for improved patient comfort and safety by employing a combined percutaneous-endoscopic rendezvous technique, which has been previously reported in adults but hitherto has not been described in children [8-13]. Successfully decompressing the biliary tree resulted in significant symptomatic, radiologic, and biochemical improvement in the patient’s condition. 

Differential diagnoses for the patient’s duodenal ulcer included inflammatory bowel disease, gastrinoma, gastritis induced by medications such as non-steroidal anti-inflammatory drugs (NSAIDs), or infectious gastritis. However, further evaluation revealed a negative inflammatory bowel disease and celiac serology, negative Helicobacter pylori IgG, IgM, and Campylobacter-like organism testing, and a normal serum gastrin level (<10). However, work-up revealed peripheral eosinophilia and elevated comparison of serum-specific (ImmunoCAP^TM^^ ^Pharmica Diagnostics AB, Uppsala, Sweden) immunoglobulin E (IgE) to shrimp (class III) and milk (class I). The patient’s hospital course was complicated by mild postoperative ERCP pancreatitis (symptoms resolved within 24 hours) and Candida isolated from his external biliary drain (potentially attributable to steroid or proton pump inhibitor use), which was treated with 200 mg of IV fluconazole daily. He was discharged on oral fluconazole 200 mg once daily and ciprofloxacin 500 mg twice daily for two weeks, sucralfate 2 mg twice daily for one month, and lansoprazole 30 mg twice daily for two months while avoiding shellfish and dairy products.

**Figure 5:**
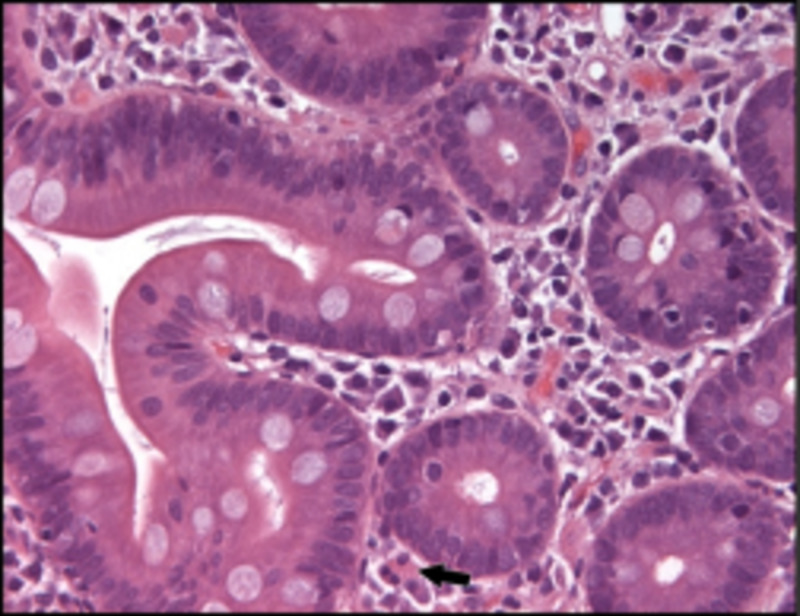
Photomicrograph of normal duodenal mucosa. There is one eosinophil in the lamina propria (bottom center, black arrow). Magnification x200.

Outpatient allergy evaluation was unremarkable, and the patient’s endoscopic stent was removed after 12 weeks with complete resolution of obstructive jaundice and cholangitis symptoms. Follow-up EGD showed a persistent eosinophilic esophagitis (>30 eos/hpf) with a small residual duodenal ulcer, and he was treated with swallowed fluticasone therapy. Repeat EGD seven months later revealed complete resolution of duodenal ulcer and duodenitis (Figure 5), with normalization of all laboratory parameters. However, eosinophilic esophagitis persisted.

Our patient presented with clinical signs and symptoms of biliary obstruction, and the diagnosis was confirmed with biomarkers, MRCP, and ERCP. EGD also revealed the presence of an inflamed duodenal ulcer just proximal to the ampulla of Vater. After fluoroscopically guided PTBD and subsequent combined percutaneous-endoscopic rendezvous drainage procedure, allergen avoidance, and appropriate medical management including systemic steroids, our patient’s biliary obstruction and duodenal disease fully resolved. 

## Discussion

EGE may employ IgE-mediated, cell-mediated, or a mixed immune pathway [8]. This may explain the variable sensitivity of IgE-mediated allergy testing, including IgE serology and cutaneous sensitivity testing, in EGE patients. If a potential food allergen is identified, then eliminating the allergen may be helpful, as was the case with our patient. Current practice for the management of EGE typically involves a six-food avoidance diet targeting the six most common triggering allergens: cow’s milk, seafood, soy, wheat, egg, and nuts [9]. Additional measures include gastric acid suppression and exogenous steroid use. In this case, selective avoidance of cow’s milk and seafood was used to avoid iatrogenic nutritional deficiency and increase ease of compliance for the patient and his family. Despite only a mild (class I) sensitivity to cow’s milk, this allergen was avoided because cow’s milk has been identified as the most common triggering food for EGE. Empiric avoidance of cow’s milk has been associated with a 65% response rate among patients with EGE [10]. 

The patient experienced significant clinical improvement and resolution of his duodenal ulcer. However, the presence of persistent eosinophilic esophagitis at seven months post-procedure despite allergen avoidance raises the possibility of ongoing exposure to an additional unidentified allergen or a primary eosinophilic disorder. Patch testing to detect non-IgE-mediated food allergens is a useful approach but was not available at our institution.

Clinical presentation of EGE depends on the region of the intestine affected and the extent of bowel wall involvement [2]. Nausea, vomiting, diarrhea, abdominal pain, poor growth, and anemia occur more commonly with eosinophilic infiltration limited to the mucosa, while obstruction may occur with submucosal and muscularis involvement [1,6]. Eosinophilic ascites and intestinal perforation are associated with serosal involvement. Despite allergen exposure, our patient’s presentation was more consistent with biliary obstruction than atopy.

Solitary duodenal ulcer formation in EGE is uncommon but not unheard of in medical literature. Markowitz et al. report a solitary ulcer as an initial presentation of EGE in a pediatric patient [1]. Additionally, two reports identify perforated duodenal ulcers in pediatric EGE patients [2,4]. Biliary obstruction associated with EGE duodenal disease is similarly rare, and until now it has only been reported in adult patients [2,7,11]. Some authors suggest that obstruction in these cases may be caused by ulcer perforation or by edema and fibrosis in the surrounding tissue [2].

Therapeutic ERCP is the endoscopic modality of choice for treating patients with obstructive biliary disease [8,11]. However, several factors, including tight biliary strictures and altered duodenal anatomy, can make this procedure challenging and lead to ERCP failure [9-12]. Combined percutaneous-endoscopic rendezvous technique is a salvage procedure following failed ERCP in which an experienced interventional radiologist performs a PTBD to temporarily relieve the biliary obstruction. A hydrophilic guidewire is then inserted through the PTBD down the biliary tree. Under fluoroscopic guidance, the wire is advanced in an antegrade fashion around the biliary obstruction and through the ampulla of Vater into the duodenal lumen, where it can be viewed endoscopically. An ERCP is then performed by an experienced therapeutic endoscopist using the wire as a guide to help ensure successful cannulation around the biliary obstruction and to promote successful downstream endoscopic biliary drainage. Combined percutaneous-endoscopic rendezvous technique has been described in several adult studies, but to our knowledge it has not been reported in the pediatric population [12-17]. 

## Conclusions

We discussed a biliary obstruction in a pediatric patient with an EGE-related periampullary duodenal ulcer. This is a severe complication of an uncommon disease and requires management of both the biliary obstruction and underlying disease process. Our patient made a full recovery after steroid treatment, allergen avoidance, and the first reported pediatric combined percutaneous-endoscopic rendezvous procedure following failed ERCP to help relieve his biliary obstruction. 
